# Supplementary figures and images for: Benznidazole treatment leads to DNA damage in Trypanosoma cruzi and the persistence of rare widely dispersed non-replicative amastigotes in mice
Source: PLoS Pathog. 2023 Nov 13;19(11):e1011627. doi: 10.1371/journal.ppat.1011627 (PMC10681306; doi:10.1371/journal.ppat.1011627)

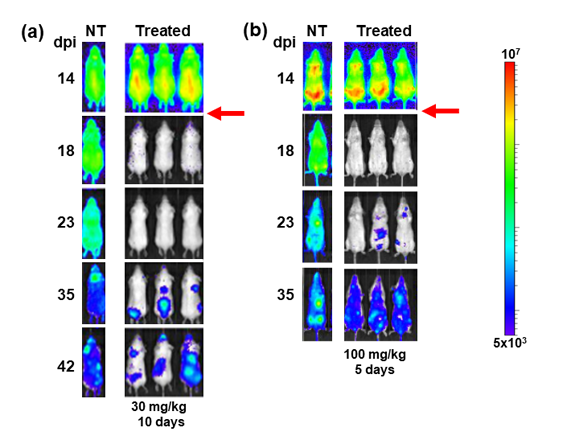

Supplement: S1 Fig — (a) Dorsal images of mice treated orally, once daily, with 30 mg/kg benznidazole for 10 days (Materials and Methods). (b) Ventral images of mice treated orally, once daily, with 100 mg/kg benznidazole for 5 days. In both instances, treatment was initiated 14 days post-infection (dpi) (indicated with red arrows). Heat-maps are on log10 scales and indicate the intensity of bioluminescence from low (blue) to high (red), with minimum and maximum radiance values as indicated. (TIF) [file ppat.1011627.s001.tif]

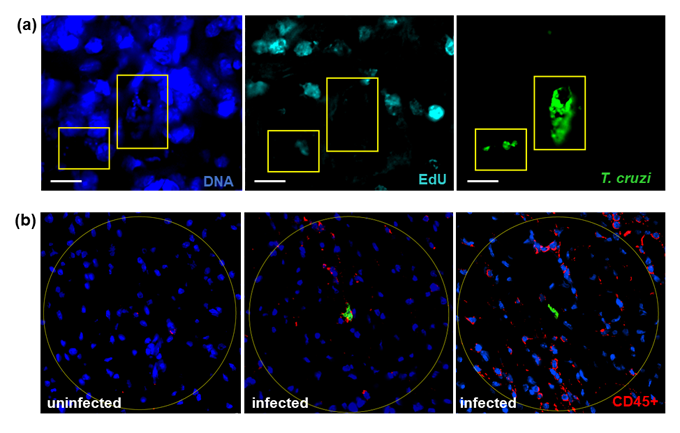

Supplement: S2 Fig — Female BALB/c mice aged 6–8 weeks were infected with T. cruzi CL Luc::mNeon (Materials and Methods). 19 dpi, the mice were given two EdU i.p. inoculations (12.5 mg/kg) 6 hours apart. They were then left overnight, euthanised, and tissue sections prepared and imaged by fluorescence microscopy (Materials and Methods). (a) Intracellular parasites in cardiac tissue imaged using a Zeiss LSM880 confocal laser scanning microscope. Note the irregular and diffuse parasite morphology. DNA (blue, DAPI); nuclei of EdU+ve host cells (turquoise); parasites (green fluorescence). White scale bar = 5 μm. (b) Sections of cardiac tissue from uninfected and T. cruzi infected mice showing infiltration of CD45+ cells (red), imaged using a Nikon Ti-2 E inverted microscope. A yellow 200 μm diameter circle is shown for reference. (TIF) [file ppat.1011627.s002.tif]

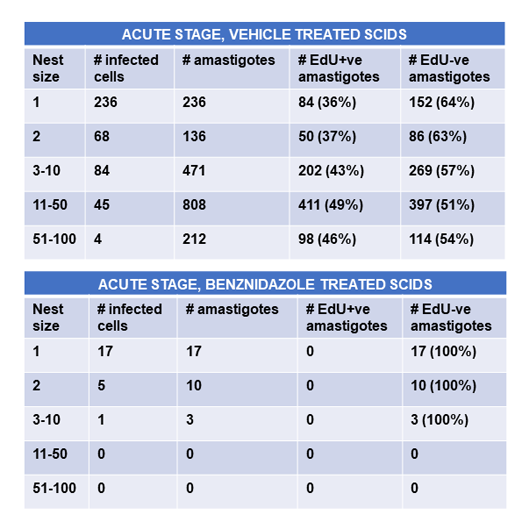

Supplement: S3 Fig — Mice were infected with T. cruzi and 10 days later treated, or not, with 5 daily doses of benznidazole (25 mg/kg). Mice were then inoculated with EdU (two doses of 12.5 mg/kg, 6 hours apart) (Materials and Methods), culled the following day, and 3 randomly selected cardiac sections from each mouse (n = 9, per group) were searched exhaustively for infected cells containing fluorescently labelled parasites. Images were acquired using a Nikon Ti-2 E inverted microscope. The numbers of EdU+ve parasites in each group are shown. (TIF) [file ppat.1011627.s003.tif]
